# Lithospheric folding by flexural slip in subduction zones as source for reverse fault intraslab earthquakes

**DOI:** 10.1038/s41598-018-19682-7

**Published:** 2018-01-22

**Authors:** I. Romeo, J. A. Álvarez-Gómez

**Affiliations:** Departamento de Geodinámica, Estratigrafía y Paleontología, Universidad Complutense de Madrid, c/ José Antonio Novais 2, 28040 Madrid, Spain

## Abstract

Subduction requires the permanent generation of a bend fold in the subducting slab which mechanics is not well understood. Lithospheric bending of subducting slabs was traditionally considered to be accommodated by orthogonal flexure, generating extensional outer rise earthquakes responsible of the external arc elongation during folding. Here we explore the possibility of lithospheric flexure being accommodated through simple shear deformation parallel to the slab (folding by flexural slip) and evaluate this process as source of earthquakes. The seismicity predicted by flexural slip dominated slab bending explains a significant amount of intermediate earthquakes observed in subduction zones with different degrees of coupling. This mechanism predicts the generation of intraslab thrust earthquakes with fault planes subparallel to the slab top. Being the orientations of the fault planes the same for the interface thrust earthquakes and the flexural-slip intraslab earthquakes, the amount of seismic moment liberated by the interface could be significantly lower than considered before. This proposed seismic source should be taken into account in models and hazard studies of subduction zones. Determining the seismic generating processes in subduction zones and their characteristics is a fundamental issue for the correct assessment of the associated seismic and tsunami risk.

## Introduction

Comprehension of deformational processes associated to subduction zones is a key issue for understanding how plate tectonics works^1–6^. The interface between the overriding plate and the subducting slab in subduction zones at shallow levels (<40 km) generates the highest magnitude and most devastating earthquakes on Earth^7–15^. Lithospheric flexure of subducting slabs was traditionally considered to be accommodated by pure shear deformation (tangential-longitudinal folding^16^, also called orthogonal flexure^17^), generating extensional outer rise earthquakes^18–21^ responsible of the external arc elongation during folding. Here we explore the possibility of lithospheric flexure being accommodated through simple shear deformation parallel to the slab (folding by flexural slip^17^) and evaluate this process as source of earthquakes. We develop three analytical geometric strain models and compare our results with observed seismicity in subduction zones: (1) an end-member model where lithospheric bending is accommodated by orthogonal flexure, (2) the other end-member model where lithospheric bending is accommodated by flexural slip and (3) a more general case where lithospheric bending is accommodated by a combination of both pure and simple shear. Finally we compare our bend fold strain models with seismicity observed in subduction zones characterized by different degrees of coupling^22^ (El Salvador, and Peru - North Chile).

The geometrical problem of bending a rigid plate has been successfully applied to the case of lithospheric bending in subduction zones reproducing the observed topography of the slab including the forebulge and outer slope trench morphology by elastic^23^, elastic-perfectly plastic^24^ and brittle-elastic-ductile models^25^. Nevertheless, subduction requires the permanent generation of a fold on the slab which internal mechanics is not well understood.

## Analytic models of slab flexure

**Figure 1:**
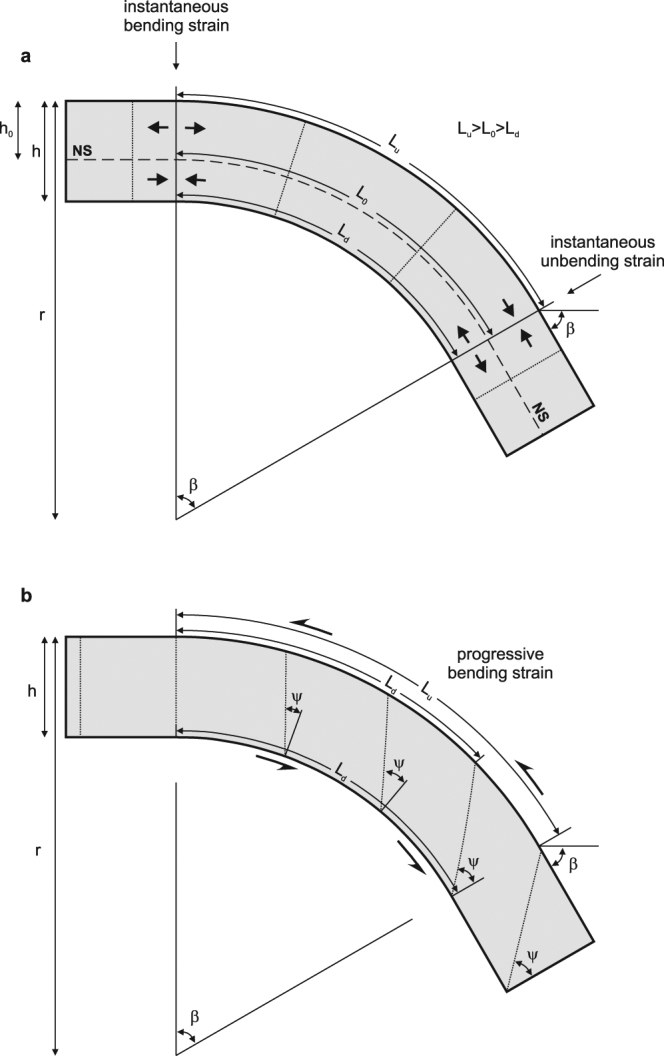
Geometrical description of the end-member flexural models for a constant curvature radius r, lithospheric thickness h and dip angle β. Dotted lines are markers initially vertical. (**a**) Orthogonal flexure: fold geometry is generated by extension of lines above and compression below NS (neutral surface), L_0_: longitude along NS which remains constant, L_u_: longitude along the top of the slab which suffers elongation, L_d_: longitude along the bottom of the slab which suffers shortening, h_0_: depth of NS. Note that markers remain as fold radius (normal to the arc) during flexure. In this model the bending strain is instantaneous when the slab enters the curved zone, and there is no strain until the slab unbends again by instantaneous strain. (**b**) Flexural flow: fold geometry is generated by conservation of longitudes of lines parallel to the slab (L) but allowing simple shear strain between these lines characterized by a shear angle (ψ). Note that, in this case, markers are curves progressively inclined during deformation. In this model, strain increases progressively with β increase.

Fold geometry implies that the longitude of the outer arc is larger than the longitude of the inner arc (Fig. 1a). When the fold is generated through orthogonal flexure, the difference of longitude of lines is accomplished by a combination of pure-shear extension of the outer arc and pure-shear compression of the inner arc. The neutral surface (NS) separates both domains, extension above and compression below, and it is characterized by a constant undeformed length. The extension suffered by the outer arc, e_u_, is given by

1 $${{\rm{e}}}_{{\rm{u}}}={{\rm{L}}}_{{\rm{u}}}-{{\rm{L}}}_{0}/{{\rm{L}}}_{0}$$

where is L_u_ the final length of the outer arc and L_0_ is the length of the neutral surface. Considering that L_u_ = β r and L_0_ = β(r − h_0_), where β is the angle described by the arc of bending in radians and is equal to the dip of the lithosphere after the arc, h_0_ is the depth of the neutral surface and r is the curvature radius, the extension of the outer arc can be expressed by

2 $${{\rm{e}}}_{{\rm{u}}}={{\rm{h}}}_{0}/({\rm{r}}-{{\rm{h}}}_{0})$$

Similarly the shortening of the inner arc, e_d_, is given by

3 $${{\rm{e}}}_{{\rm{d}}}=({{\rm{L}}}_{{\rm{d}}}-{{\rm{L}}}_{0})/{{\rm{L}}}_{0}$$

where L_d_ is the final length of the inner arc. Considering that L_d_ = β (r − h) where h is the lithospheric thickness and L_0_ = β(r − h_0_) the shortening of the inner arc can be expressed by

4 $${{\rm{e}}}_{{\rm{d}}}=({{\rm{h}}}_{0}-{\rm{h}})/({\rm{r}}-{{\rm{h}}}_{0})$$

From Eqs 2 and 4 can be deduced that the only parameters that control the amount of deformation necessary to generate the bending are: r, the radius of curvature; h, the lithospheric thickness; and h_0_, the depth of the neutral surface. The amount of deformation does not depend on the dip of the lithosphere (β) therefore it is independent on the position in the bend fold. When a portion of the subducting lithosphere enters the bend zone it suffers an instantaneous pure shear deformation which provides the arched geometry with the specific curvature radius of that subduction zone. When this portion of the lithosphere passes through the bend it does not suffer any new deformation related with the flexure, except if there is a change of the curvature radius. When it leaves the bend it undergoes the same amount of pure shear deformation in the opposite sense (compression above and extension below the neutral surface) in order to restore the flat original geometry of the slab.

The simple-shear model (Fig. 1b) considers that there is no extension or compression of lines parallel to the lithosphere, but shear slip between them is allowed. The amount of shear deformation is defined by the shear angle (ψ),

5 $${\rm{\Psi }}=\arctan ({{\rm{L}}}_{{\rm{u}}}-{{\rm{L}}}_{{\rm{d}}}/{\rm{h}})$$

This expression can be simplified when it is combined with L_u_ = β r and L_d_ = β (r − h), providing:

6 $${\rm{\Psi }}=\arctan ({\rm{\beta }})$$

Therefore, the only parameter that controls the amount of deformation in the simple-shear model is the dip of the flexed lithosphere. In this case, deformation takes place gradually with the growing of the dip angle. When a portion of lithosphere leaves the bend zone, it remains deformed by simple-shear and does not suffer any new deformation.

Slip rates (ė) for flexural slip in the brittle domain (total displacement from the slab top to the limit between the brittle and elastic domains) can be calculated by

7 $$\dot{{\rm{e}}}={\rm{v}}({{\rm{Z}}}_{{\rm{b}}}/{\rm{r}}-{{\rm{Z}}}_{{\rm{b}}})$$

where v is the velocity of plate convergence, Z_b_ is the thickness of the brittle domain of the lithosphere and r the curvature radius.

A combination of both flexure mechanisms could be a more realistic approximation to the problem. For this case, both mechanisms do not take place at the same location nor at the same moment. Part of the curvature can be accommodated by orthogonal flexure at bend onset producing outer-rise normal faulting. Flexural slip requires the accumulation of strain with the raising dip angle to generate reverse faulting parallel to the slab top. Therefore the combination model have two tectonic stages overprinted, first orthogonal flexure generating part of the curvature and later a superposed flexural slip stage providing a flexure with the final curvature.

## Method

The application of our analytic models to real subduction zones was performed using the following procedure. The geometry of the top slab surface was obtained from Slab 1.0 model^26^. Each subduction zone was divided into several sectors with constant curvature radius (r). The limits between the sectors were obtained tracing perpendicular lines to the top slab surface. El Salvador subduction zone was divided into three sectors: the first sector mainly located in the trench outer wall is characterized by a low curvature (r = 576 km), the second sector were the slab gets its maximum curvature (r = 172 km) and the last sector were the slab goes deeper and loosing curvature (r = 463 km). The slab geometry of the Peru subduction zone was divided in two sectors with the same low curvature (r = 447 km) but opposite sense. Between both sectors there is a small zone (30 km wide) with a dominant planar geometry where curvature inverts from convex to concave.

Orthogonal flexure requires an estimation of the depth of the neutral surface which was obtained through a brittle-elastic-ductile lithospheric model calculating Yield Strength Envelopes (YSEs) in lines perpendicular to the slab. For the construction of YSEs the half space cooling model^27^ was assumed to be a good approach for the thermal profile considering the relatively young oceanic lithospheres modelled (El Salvador 26 Myr and Peru 50.5 Myr). Brittle domain was modelled using the equations for frictional sliding sensitive only to pressure (depth). A dry olivine flow law^25^ was used to model ductile mantle deformation. The bending stress profile is calculated according to the curvature of the slab at each point. Neutral surface depth is computed integrating the stresses of the brittle, elastic and plastic domains, equaling the bend momentum supported by the upper tensional domain and the lower compressional domain^25^. No external stresses, including ridge push, trench pull or mantle drag forces were added to our model since we want to constrain the deformation associated exclusively to flexure. This procedure allows us to calculate what proportion of the total deformation is caused by flexure. Our geometrical analytical models do not allow to calculate what portion of the strain generated by bending is accommodated by orthogonal flexure or flexural slip. This calculation is strongly dependent on the mechanical structure of the lithosphere specially on the degree of stratification which, in the brittle domain, is highly sensitive to water release through dehydration metamorphic reactions and migration along discontinuities leading to water overpressure embrittlement.

### Application of the models to subduction zones

**Figure 2:**
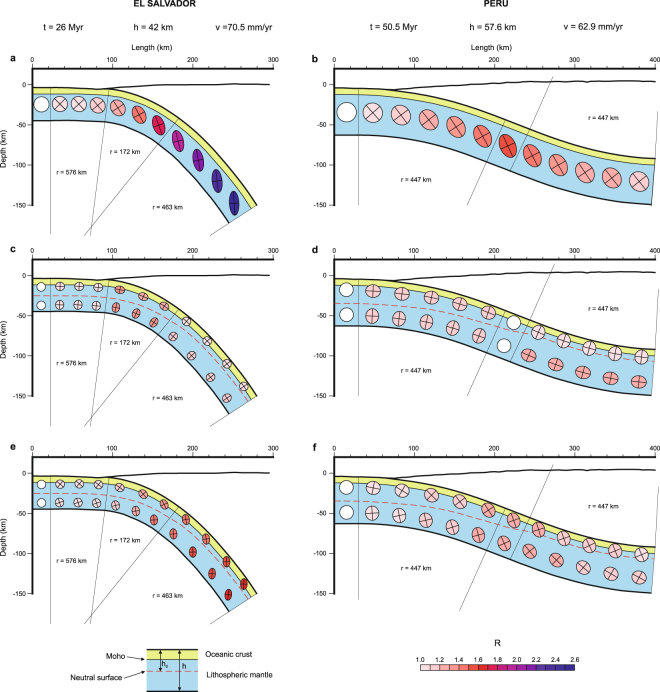
Flexural models applied to real subduction zones on El Salvador (**a**,**c**,**e**) and Peru - North Chile (**b**,**d**,**f**). Two end member models based on flexural flow (**a**,**b**) and orthogonal flexure (**c**,**d**) are shown. A more general model (**e**,**f**) generated by 50% of orthogonal flexure and 50% of flexural flow is also shown. Note how strain increases with dip in flexural flow models (**a**,**b**). Ellipses correspond to finite strain ellipses of the bulk deformation accumulated from the beginning of subduction to each point. Note that for Peru the change from convex to concave geometry produces tectonic inversion. Plane deformation and mass conservation were assumed. Dashed red line corresponds to the neutral surface. Strain shown by ellipses correspond to the top and bottom of the slab, although the were relocated inside the slab improving figure clearness. The amount of cumulated strain is indicated by R, the ratio between the long and short strain ellipse axes.

**Figure 3:**
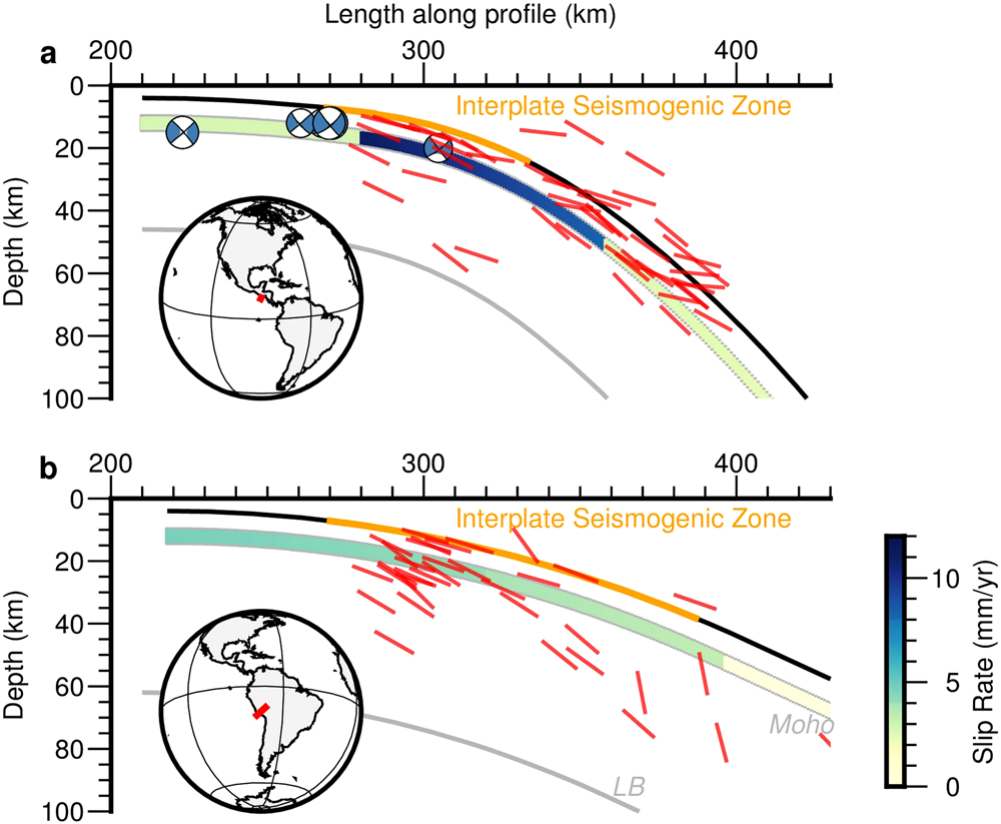
Reverse faulting earthquakes on (**a**) El Salvador and (**b**) Peru - North Chile subduction. Inset shows profile locations for the projection of earthquakes on a 100 km wide band. Reverse earthquakes arc-dipping nodal planes are plotted as red lines. Note the abundance of reverse fault earthquakes with nodal planes oriented parallel to the slab, coincident with the expected flexural slip bending planes, specially concentrated at Moho surface in the zone where strong curvature and high slab dip are reached. Slip rate in the brittle domain calculated from flexural flow model Eq. (7) is also shown. Outer-rise normal faulting earthquakes are shown as focal mechanisms, not present in Peru – North Chile subduction. LB: Lithospheric base. Focal mechanisms from the Global CMT catalog^32^.

We have applied the proposed 2D flexural models of deformation to real subduction zones (Fig. 2): 1) Central American subduction in El Salvador, characterized by high slab dip and low coupled subduction interface which extends to a depth of 25 km^28,29^ and 2) Peru - North Chile, featuring low slab dip and coupled subduction interface to a depth of 40 km^30,31^. Flexural-slip (Fig. 2a,b), orthogonal flexure (Fig. 2c,d) and a combination of 50% orthogonal flexure and 50% of flexural-slip, Fig. 2e,f) models were applied to both subduction zones in order to compare model deformation with current seismicity (Fig. 3) with the aim of unravel the active mechanisms of internal slab deformation due to flexure.

On the one hand, the extensional outer rise earthquakes of El Salvador indicate a component of pure-shear deformation during flexure, which rules out the flexural model only accomplished by simple-shear (Fig. 2a), although a component of simple-shear can be present (Fig. 2e) or not (Fig. 2c). On the other hand, the mechanical coupling of the Peru subduction interface could prevent extensional outer rise earthquakes to occur. In this case, since there is no evidence of pure-shear extension by brittle deformation in the outer arc, flexure could be mainly accommodated by simple-shear. The short time span of the seismic catalog regarding rupture mechanisms prevents us of completely ruling out some pure shear component of the deformation in the Peru subduction, as the outer rise seismicity seems to be frequent on most of subduction zones^21^.

Pure-shear extension along slab in the outer arc is calculated by Eq. 2 and pure-shear shortening along slab in inner arc is calculated by Eq. 4. The strain along the axis perpendicular to the slab surface is calculated assuming volume conservation (area conservation in our 2D model), this assumption implies plane deformation (no deformation is allowed perpendicular to the 2D model). Strain ellipses for the models that combine both mechanisms (Fig. 2e,f) were calculated producing first the pure-shear component of deformation and later the simple-shear one, because orthogonal flexure requires an instantaneous deformation when the slab enters the bend zone, while flexural-slip deformation increases gradually with dip thus it takes place later. Each strain ellipse of Fig. 2 represents the finite total deformation accumulated due to slab flexure until the subducted material reached that point. In the case of Peru (Fig. 2b,d,f) the deformation associated to the initial convex flexure is later inverted by a final concave flexure.

### Comparison with seismicity and discussion

**Figure 4:**
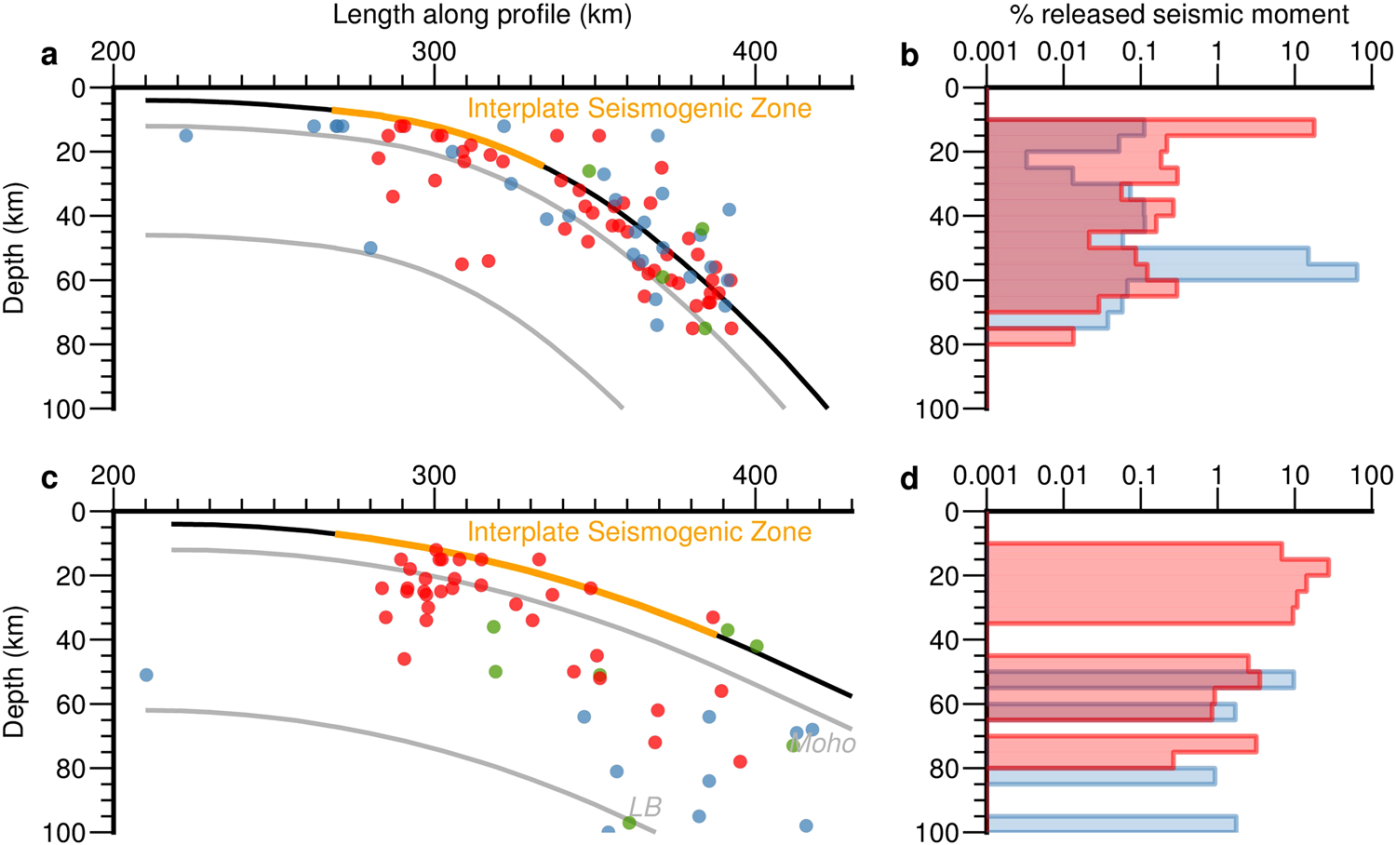
Projected earthquake locations classified as normal (blue), reverse (red) and strike slip (green) dots for (**a**) El Salvador and (**c**) Peru - North Chile subductions. The interplate seismogenic zone is shown as an orange thick line. The amount of seismic moment released by normal and reverse ruptures per depth is expressed as percentage of the total seismic moment for (**b**) El Salvador and (**d**) Peru - North Chile. LB: Lithospheric base. Focal mechanisms from the Global CMT catalog^32^.

Figure 3 shows outer rise normal fault focal mechanisms (only present in El Salvador) related to orthogonal flexure and all the thrust faults earthquake nodal planes dipping towards the volcanic arc (focal mechanisms from the Global CMT catalog^32^). The expected rupture mechanism of earthquakes for convex curvature flexural slip is reverse faulting with the rupture plane parallel to the slab top surface. Figure 3 shows that almost all intraslab thrust earthquakes are oriented as flexural slip predicts (a nodal plane sub-parallel to the slab top). The earthquake cloud dominated by reverse faults has been traditionally associated almost exclusively to the subduction interface, but 73% of the thrust earthquakes in El Salvador and 25% in Peru are located below the lower limit of the interplate seismogenic zone. Moreover, taking into account location uncertainties, the earthquakes located in the Moho and below probably represent intraslab deformation caused by flexural slip even at interplate seismogenic zone depths. Figure 4 shows all the seismicity for each subduction zone classified as normal, reverse or strike-slip events indicating the amount of released seismic moment with depth expressed as percentage of the total seismic moment (focal mechanisms from the Global CMT catalog^32^). Thrust fault events bellow the interplate seismogenic zone represent 2% for El Salvador and 42% for Peru of the total seismic moment released at this depth (61% of the number of events for El Salvador and 43% for Peru). While the seismicity that can be caused by orthogonal flexure is limited to only 6 normal outer rise earthquakes in El Salvador and 0 events in Peru, flexural slip provides a successful explanation for a long number of events in both subduction zones (Figs 3 and 4).

Classically the intermediate intraslab seismicity has been related to slab-pull forces producing tensional earthquakes and unbending forces generating compressive earthquakes. These earthquakes form frequently a double Wadati - Benioff zone^33^ with the former producing the lower zone and the latter the upper one^34^, being both driven by fluid released in dehydratation reactions^35,36^. Despite this model is able to explain the location of the seismicity and the causes of slab enbrittlement, the mechanism driven the slab deformation is still mater of debate^36^ with the spatial coexistence of down-dip extension focal mechanisms and down-dip compression, or reverse faulting, focal mechanisms. The classical model of pure-shear deformation (membrane stresses by bending and unbending) fails to explain completely the observed seismicity and compressional forces from the upper plate are usually wielded to explain them. These earthquakes can be explained also if a component of flexural slip is present.

Slip rates expected by flexural slip folding were calculated along subduction profiles (Fig. 3) using Eq. 7. From these slip rates we can estimate the potential seismic moment release (assuming μ = 45 GPa) which is 4.9 × 10^18^ Nm/yr for El Salvador and 3.3 × 10^18^ Nm/yr for Peru. These results suggest that flexural slip bending has enough seismic potential to generate a M_W_ 6.4 magnitude event in El Salvador and M_W_ 6.3 magnitude event in Peru per year per 100 km along trench. Obviously the rheology of the subducted slab is far from being a perfectly elastic material with brittle behaviour, and consequently a great part of the deformation must be accommodated aseismically.

Flexural slip is favored by discontinuities parallel to the folded layer, although their presence is not a necessary condition for flexural slip to take place^37^. Oceanic lithosphere has been traditionally considered a mechanically homogeneous layer for flexural modelling purposes, supporting orthogonal flexure as the main bending mechanism. Nevertheless it is well known that this homogeneity is an oversimplification, since there is a main mechanical discontinuity at the Moho and also there are other minor discontinuities between the layers of the oceanic crust, providing an anisotropy parallel to the slab top in the brittle domain of the lithosphere. In detail, the Moho shows complexities like the differences between a deeper petrological Moho (between lithospheric mantle and ultramafic cumulates) and a shallower seismic Moho (between ultramafic cumulates and mafic cumulates), being the last one more important as mechanical discontinuity. The contacts between seismic crustal layers 3B (mafic cumulates), 3A (massive gabbro, diorite and plagiogranite and the mafic sheeted dike complex on top), 2 (mafic extrusives: pilowlavas and massive flows) and 1 (sediments) are other remarkable mechanical discontinuities present in the oceanic crust^38^. Moreover, looking at a detailed scale, all the mafic and untramafic cumulates have an strong anisotropy subparallel to the slab top provided by the magmatic layering generated in the magma chamber at the ridge.

**Figure 5:**
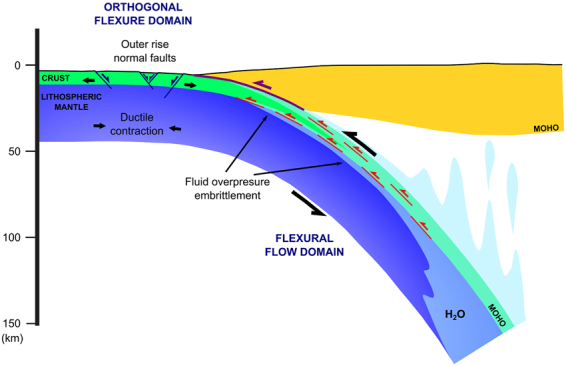
Main deformation domains of strain related to flexure for both mechanisms orthogonal flexure and flexural flow. Note the role of ascending water from dehydration reactions along the Moho and the slab top causing embrittlement where intraslab seismicity is observed. Outer rise normal faults (blue) and flexural slip related reverse faults (red) are also shown.

Recent studies have shown the relevance of Moho as a path for water raising from dehydration reactions of the underlaying lithospheric mantle^39–42^ and its relevance on intraslab seismicity^43,44^. Water overpressure at Moho surface promotes faulting at the zone where simple-shear flexural earthquakes are expected to take place (Fig. 5). Although simple-shear flexural earthquakes are mainly expected to take place at the base of the crust, other parallel discontinuities can nucleate faulting as well. Double Seismic Zones on intermediate earthquakes on subduction zones can also reflect flexural slip deformation, explaining why some seismic clusters are not clearly related to down-dip extension or compression zones^36^. Observed swarms aligned on the slab Moho in shallow seismicity in the low coupled subduction in Central America^45^ as well as compressive shallow intraslab earthquakes^46^ can also be explained by this mechanism.

Lithospheric flexure in highly coupled subduction zones is controlled by the seismic cycle of the main interface thrust earthquakes. During interseismic periods elastic deformation is accumulated in the sides of the blocked plate interface while subduction and consequently progressive lithospheric flexure is going on at depth. The blocked behavior of the interface during this period prevents the stresses associated to flexure to be transmitted to the outer rise avoiding normal faulting related to orthogonal flexure to take place. This promotes the accommodation of lithospheric flexure during the interseismic period, which represents most of the seismic cycle, by flexural slip. The unlocking of the interface accomplished coseismically with large interplate events allows flexural stresses to be transmitted to the outer rise and the generation of normal faulting associated to orthogonal flexure in this area during the postseismic period. Outer rise normal faulting triggered by main subduction thrust earthquakes has been observed for example in Kuril Islands^47^, Chile^48^ and Japan^49–51^. Thus, lithospheric flexure in coupled subduction zones, could oscillate from flexural slip to orthogonal flexure controlled by the seismic cycle.

Lithospheric bending through flexural slip on subduction zones could have implications in the processes involved in complex subduction dynamics revealed by studies of ancient subduction zones^52,53^. If flexural slip is mainly accommodated by faulting along the Moho or other parallel crustal discontinuities, the decoupling of the oceanic crust from the lithospheric mantle or the decoupling between crustal units could facilitate the detachment of units at depth in subduction zones.

## Conclusion

Flexural slip as a mechanism of slab bending in subduction zones provides a satisfactory explanation for a large number of intraslab thrust earthquakes with fault planes parallel to the slab top. This seismicity is present in subduction zones with different slab dip angle and degree of coupling. This proposed seismic rupture process should be taken into account in future studies on subduction models as well as in tsunami and seismic hazard assessments.
